# Effectiveness of leuprolide acetate administered monthly compared to three-monthly in the treatment of central precocious puberty: evaluation at the end of treatment

**DOI:** 10.3389/fendo.2024.1390674

**Published:** 2024-04-26

**Authors:** Thanaporn Thaneetrakool, Suphab Aroonparkmongkol, Nattakarn Numsriskulrat, Vichit Supornsilchai, Suttipong Wacharasindhu, Khomsak Srilanchakon

**Affiliations:** ^1^ Division of Pediatric Endocrinology, Department of Pediatrics, Faculty of Medicine, Chulalongkorn University, Bangkok, ;Thailand; ^2^ Division of Academic Affairs, Faculty of Medicine, Chulalongkorn University, Bangkok, ;Thailand

**Keywords:** central precious puberty, leuprolide acetate, effectiveness, 3-monthly formulation, monthly formulation

## Abstract

**Introduction:**

Gonadotropin-releasing hormone (GnRH) analogs are the standard treatment for central precocious puberty (CPP). Although there are numerous varieties of GnRH agonists, the effectiveness of 1-monthly compared with 3-monthly Leuprolide acetate is still restricted. The objective of this study was to evaluate the outcomes of CPP treatment with Leuprolide acetate at a 1-monthly dosage of 3.75 mg, in comparison to a dosage of 11.25 mg administered every 3 months.

**Method:**

This retrospective cohort study involved 143 girls diagnosed with CPP with 72 of them receiving the monthly treatment regimen and 71 receiving the 3-monthly treatment regimen. Anthropometric measurements were compared at the start and end of the therapy. The rates and level of LH suppression were assessed six months after therapy.

**Results:**

The regimen administered every 3 months showed more significant suppression of LH. The 3-monthly group showed lower actual height and degree of bone age advancement at the end of therapy. However, the predicted adult height (PAH) remained comparable in both groups.

**Conclusion:**

The 3-monthly treatment showed greater hormonal and growth suppression effects, but there was no significant difference in PAH between the two groups.

## Introduction

Central precocious puberty (CPP) develops when the hypothalamic-pituitary-gonadal (HPG) axis is activated prematurely at an age below eight years old in girls (1). The condition can occur because of many defects, however in the majority of cases, its cause remains unidentified (1, 2). Idiopathic cases are considerably more prevalent in girls, accounting for approximately 90% among girls (2). The diagnosis of CPP in girls is associated with numerous problems such as early menarche, low adult height due to early epiphyseal fusion, and negative psychosocial effects (1). Therefore, it is important to provide appropriate therapy to girls with CPP for the purpose avoid these negative consequences.

Gonadotropin releasing hormone (GnRH) analogs are standard of care for treatment of CPP (3). GnRH analogs bind to the pituitary GnRH receptors continuously, limit the pulsatile secretion of gonadotropins which leads to pubertal activation. The mechanism of action involves the downregulation of the GnRH receptors and subsequent suppression of HPG axis (4, 5). Optimal treatment results in stabilization of pubertal progression, a decline of growth velocity, a decrease of bone age advancement, and increase final adult height (3, 6, 7).

Nowadays, there are several GnRH analog preparations available. The 1-monthly depot formulation has been the primary option for treating CPP since the mid-eighties (8, 9). The 3-monthly depot formulation was later introduced and has demonstrated its capability to effectively suppress the pituitary-gonadal axis and pubertal development (10–12). There is a limited number of studies comparing the effectiveness of these two formulations, particularly at the end of treatment. There was a study that compared adult height in 25 girls with CPP who received triptorelin acetate monthly and triptorelin acetate every three months. The study found no significant difference in their final height (13).

In this study, we examined the outcomes of Thai girls with CPP who were treated with a Leuprolide acetate at a dosage of 3.75 mg every month and compared it with a dosage of 11.25 mg every 3 months, to assess their effectiveness in suppressing the HPG axis.

## Materials and methods

### Participants

A total of 143 girl participants diagnosed with CPP were included at the pediatric endocrinology clinic of King Chulalongkorn Memorial Hospital from December 2008 to December 2022. The inclusion criteria comprised girls with CPP, defined as the onset of breast development before the age of 8, along with a hormonal profile showing baseline LH > 0.3 IU/L or peak LH after a GnRH stimulation test > 5 IU/L, who underwent treatment with Leuprolide acetate. MRI scans were provided for some patients, particularly those with an age of breast onset before 6 years old. The treatment involved either a monthly dosage of 3.75 mg or a 3-monthly dosage of 11.25 mg intramuscularly. Exclusion criteria included individuals with a history of external hormonal use, congenital adrenal hyperplasia, and growth hormone deficiency.

### Methodology

This retrospective cohort study gathered data at the time of diagnosis and during follow-up visits. The collected data included chronological age (years), age of breast onset (years), weight (SDS), height (SDS), body mass index (BMI) (SDS), midparental height (MPH) (cm), breast Tanner stage, menarche status, bone age (years), and predicted adult height (PAH) at the initiation of treatment (cm, SDS). SDS values for weight, height, and BMI were determined using the World Health Organization (WHO) growth curve. The pediatric endocrinologist utilized the Greulich and Pyle technique for bone age assessment, and PAH was calculated using the Bayley-Pinnuau method (14).

### LH suppression

The effectiveness of leuprolide acetate treatment was assessed through the LH suppression at 6 months post-treatment. Serum LH levels were obtained 2 hours after the administration of the treatment dose of leuprolide acetate (3.75 mg or 11.25 mg) and measured utilizing Electrochemiluminescent Immunoassay (ECLIA). LH suppression was defined as serum LH levels falling below 4 IU/L (1, 4).

### Growth parameter

The growth outcomes after treatment were evaluated at the end of therapy, with a focus on height at treatment completion. This included height at the end of treatment (SDS), degree of bone age advancement, PAH at the end of treatment, the difference between PAH at the start of treatment and PAH at the end of treatment (cm), and the difference between PAH at the end of treatment and MPH (cm).

### Ethical consideration

This study constitutes a retrospective analysis conducted through the review of medical records and does not involve an examination of the treatment decisions made by physicians. Approval for the study was obtained from the Institutional Review Board of the Faculty of Medicine, Chulalongkorn University (IRB No.0726/66), in accordance with international guidelines for human research protection, including the Declaration of Helsinki, The Belmont Report, CIOMS Guideline, and International Conference on Harmonization in Good Clinical Practice (ICH-GCP).

### Statistical analysis

Statistical analyses were executed using SPSS (version 29.0). Continuous variables, both with and without normal distribution, were reported as mean (standard deviation) and median (interquartile range), respectively. Categorical data were presented as proportions (percentage). The independent t-test was employed to assess differences in continuous variables between the two groups, while the Chi-square test was utilized to analyze differences in categorical variables. A p-value of <0.05 was considered statistically significant.

## Result

A total of 143 girls participated in the study, with 72 receiving monthly treatment of Leuprolide acetate 3.75 mg and the remaining 71 girls receiving treatment with Leuprolide acetate 11.25 mg every three months. The baseline clinical characteristics and hormonal profiles at the start of treatment are presented in **Table 1**. Demographic data, including age at the start of treatment, age of onset, auxological data, bone age, and PAH were found to be similar in both groups without any significant differences. From the MRI scans, it was observed that 15 patients had pituitary lesions, with 8 patients in the monthly group and 7 patients in the three-monthly group. All patients are administered leuprolide acetate until their bone age reaches approximately 12 years, with the treatment duration around 2 years (13).

**Table 1:** Demographic data.

	Leuprolide acetate		
	1 monthly (N = 72)	3 monthly (N = 71)	P-value
	Mean ± SD	Mean ± SD	
Age of onset, years	7.13 ± 1.1	7.34 ± 0.7	0.16
Weight, kg	34.71 ± 8.6	33.03 ± 8.7	0.25
Weight, SDS	1.59 ± 1.1	1.30 ± 1.3	0.12
Height, cm	134.93 ± 9.0	134.46 ± 8.2	0.74
Height, SDS	1.66 ± 1.0	1.52 ± 1.2	0.46
BMI, kg/m^2^	18.81 ± 3.1	17.99 ± 3.0	0.11
BMI, SDS	1.09 ± 1.3	0.75 ± 1.3	0.11
MPH, cm	156.94 ± 4.4	157.78 ± 4.6	0.28
Bone age, years	10.71 ± 1.6	10.48 ± 1.5	0.37
PAH-av, cm	150.98 ± 6.3	152.51 ± 7.4	0.19
PAH-av, SDS	-1.26 ± 1.4	-0.94 ± 1.6	0.19
PAH-ac, cm	155.42 ± 6.9	157.15 ± 7.9	0.17
PAH-ac, SDS	-0.32 ± 1.5	0.04 ± 1.7	0.17
BA-CA	2.50 ± 1.1	2.22 ± 1.1	0.15
BA/CA	1.31 ± 0.2	1.27 ± 0.1	0.13

SDS, standard deviation score; BMI, body mass index; MPH, midparental height; PAH, predicted adult height; BA, bone age; CA, chronological age.

Following 6 months of treatment, LH suppression was assessed and presented in **Table 2**. The mean LH level was observed to be higher in the 1-monthly treatment group (2.7 ± 1.9 and 1.9 ± 0.7, p-value 0.049), and this difference was statistically significant. Additionally, the LH suppression rate was lower in the 1-monthly treatment group (84.7% and 100%, p-value 0.06).

**Table 2:** LH Suppression at 6-month of treatment.

	Leuprolide acetate		
	1 monthly	3 monthly	p-value
	Mean ± SD	Mean ± SD	
Mean LH level (IU/L)	2.72 ± 1.8	1.86 ± 0.7	0.049*
% LH < 4 IU/L	84.7%	100%	0.06
Mean Estradiol level (pg/mL)	26.3427.65	7.064.26	<0.001

SDS, standard deviation score; LH, luteinizing hormone.

*p < 0.05.

**Figure 1:**
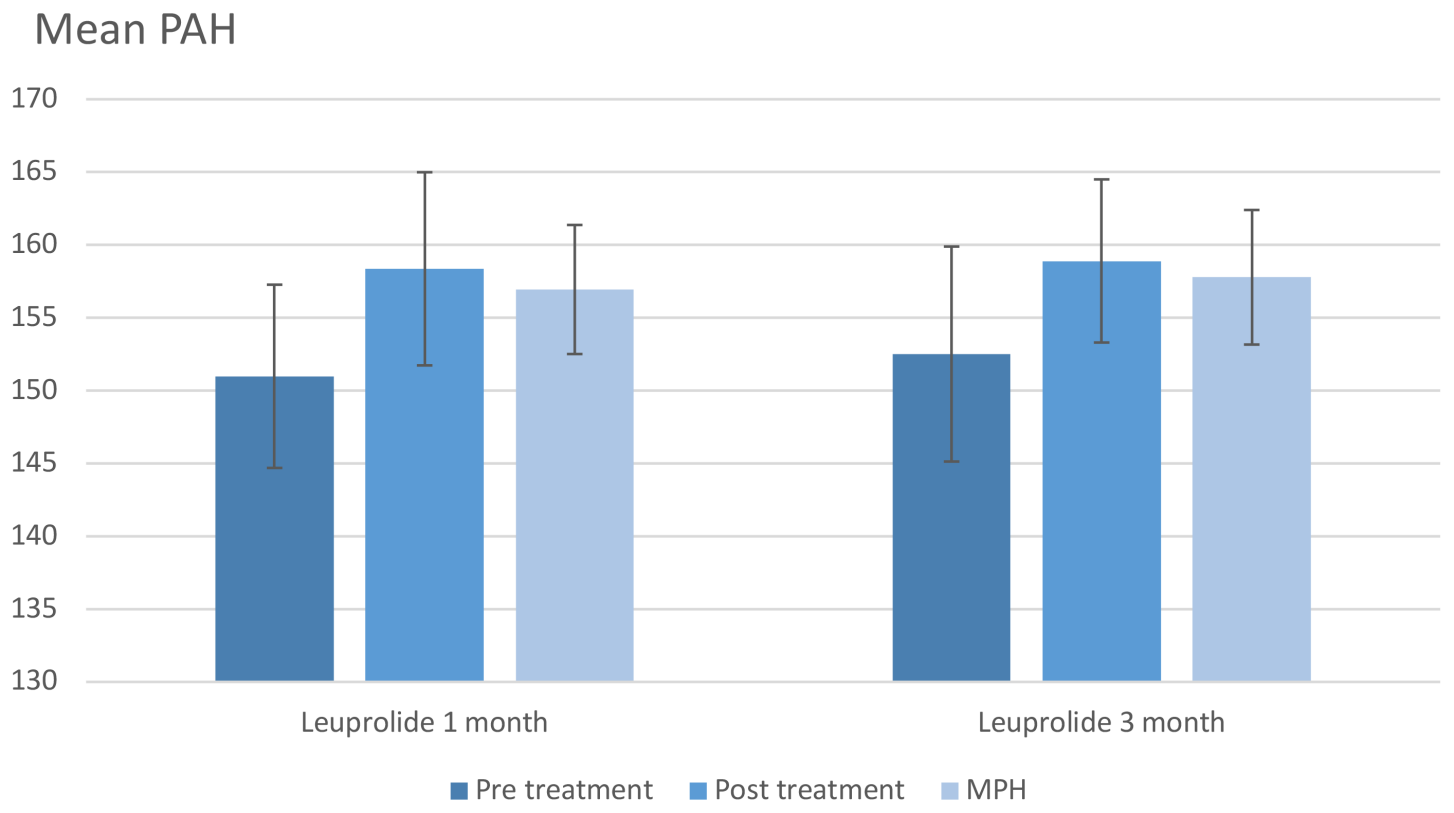
Predicted adult height (average). MPH, midparental height; PAH, predicted adult height.

The growth parameters at the end of treatment are depicted in **Table 3**. The degree of bone age advancement, represented by BA minus CA, was higher in the 1-monthly group and reached statistical significance (1.49 ± 0.9 and 0.88 ± 2.1, p-value 0.049). Upon comparing PAH at the end of treatment with PAH at the start of treatment, both groups exhibited an increase in PAH at the end of treatment compared to the start of treatment. Notably, the 1-monthly group showed a significant rise in PAH compared to the 3-monthly group (6.58 ± 5.5 and 4.19 ± 4.6, p-value 0.026). **Figure 1** illustrates PAH comparison between pretreatment, post-treatment, and MPH, demonstrating improved PAH after treatment in both groups and approaching near MPH.

**Table 3:** Growth parameter at end of treatment.

	Leuprolide acetate		
	1 monthly	3 monthly	p-value
	Mean ± SD	Mean ± SD	
Height			
Height last treatment, cm	147.64 ± 6.3	145.12 ± 7.7	0.07
Height last treatment, SDS	1.00 ± 1.1	1.04 ± 1.1	0.85
BA/CA			
0 month	1.31 ± 0.2	1.27 ± 0.1	0.13
End of treatment	1.14 ± 0.1	1.09 ± 0.2	0.07
BA-CA			
0 month	2.50 ± 1.1	2.22 ± 1.1	0.15
End of treatment	1.49 ± 0.9	0.88 ± 2.1	0.04*
PAH av			
0 month, cm	150.98 ± 6.3	152.51 ± 7.4	0.19
0 month, SDS	-1.26 ± 1.4	-0.94 ± 1.6	0.19
End of treatment, cm	158.36 ± 6.6	158.90 ± 5.6	0.67
End of treatment, SDS	0.29 ± 1.4	0.41 ± 1.2	0.67
PAH_end_ – PAH_start_	6.58 ± 5.5	4.19 ± 4.6	0.03*
PAH ac			
0 month, cm	155.42 ± 6.9	157.15 ± 7.9	0.17
0 month, SDS	-0.32 ± 1.5	0.04 ± 1.7	0.17
End of treatment, cm	161.58 ± 7.2	163.09 ± 5.8	0.27
End of treatment, SDS	0.97 ± 1.5	1.29 ± 1.2	0.27
PAH_end_ – PAH_start_	5.27 ± 6.2	3.30 ± 5.6	0.11
MPH			
PAH av-MPH	1.59 ± 5.3	1.97 ± 5.2	0.74
PAH ac-MPH	4.81 ± 5.9	6.09 ± 5.5	0.31

SDS, standard deviation score; BA, bone age; CA, chronological age; PAH, predicted adult height; PAH_end_, Predicted adult height at end of treatment; PAH_start_, Predicted adult height at start treatment; MPH, midparental height.

*p < 0.05.

## Discussion

In the treatment of girls with CPP in Thailand, two dosage regimens of GnRH agonists are available, but studies comparing the effectiveness of these regimens remain limited. From the previous study (15), the LH suppression level was similar in both the leuprolide 3.75 mg monthly group and the 11.25 mg three-monthly group after 12 weeks of treatment. However, in the study comparing leuprolide 11.25 mg and 30 mg of leuprolide acetate every 3 months, higher suppression was observed with the higher dose (16).

Initially, we assessed the effectiveness through investigating LH suppression after 6 months of treatment, since recent studies and our study have shown that both dosage regimens may successfully reduce LH levels after 6 months of treatment (17, 18). The result revealed greater suppression in the 3-monthly group. The mean LH levels in both groups were below 4, but they were lower in the 3-monthly group, with a 100% LH suppression rate observed in this group. The results of this study are consistent with previous research conducted in China (19), which found that LH suppression rates were 100% in the 3-monthly group and 95% in the 1-monthly group. Prior studies on the pharmacokinetics of both regimens conducted for prostate cancer treatment, showed higher peak levels and a longer time to decrease to steady state in the 3-monthly group. This may contribute to the greater suppression in the 3-monthly group. Nevertheless, when comparing concentration at steady state and median tough levels, both groups exhibited similar results, and there was no significant difference in suppressing testosterone levels (20, 21).

Previous studies have investigated growth outcomes after 1 year of treatment, revealing no significant differences in height, height velocity, and the degree of bone age advancement between both groups (19, 22, 23). However, there is limited information regarding the evaluation of the effectiveness of these medications through the assessment of growth parameters after the completion of therapy. Our study aimed to provide additional insights into long-term growth outcomes. We observed that the actual height of the 3-monthly group was slightly lower than that of the 1-monthly group. When assessing the degree of bone age advancement, there was more advancement in the 1-monthly group than in the 3-monthly group, as determined by both BA divided by CA and BA minus CA. These findings align with the results of LH suppression. The differences in bone age advancement observed in our study may be attributed to the longer treatment duration compared to previous studies.

When the PAH at the end of treatment was compared to its value at the start of treatment, a greater difference was observed in the 1- monthly group. This may seem counterintuitive, especially considering the more advanced bone age in the 1-monthly group. We hypothesized that the lesser PAH difference in the 3-monthly group might be due to lower actual height in this group. Considering the lower actual height at the end of treatment in the 3-monthly group, it suggests that the 3-monthly group is more effective in suppressing growth, resulting in a lower actual height. This observation prompts the consideration that, while the 3- monthly group is more effective in suppressing the advancement of bone age, it also suppresses actual height. In the end, there were no differences in PAH, aligning with a previous study that evaluated final height in girls treated with 1-monthly and 3-monthly regimens of triptorelin, where no significant different in final height were observed (13).

The limitations of our study include its retrospective nature, leading to some missing data and variations in the number of data points for certain parameters, making it challenging to compare between the two groups. It is important to acknowledge that we did not assess the actual final adult height because, in the 3-monthly group, the treatment began in 2019, and all patients in this group have not reached their final adult height yet. Despite these limitations, our study provides valuable insights into the comparative effectiveness of the two Leuprolide acetate regimens in the treatment of CPP.

## Conclusion

In the treatment of girls with CPP using 1-monthly and 3-monthly regimens of leuprolide acetate, the 3-monthly regimen demonstrates greater effectiveness in LH suppression and in suppressing bone age advancement. Both regimens can increase PAH after treatment; however, there are no significant differences in PAH between the two regimens. Further long-term prospective studies to assess actual final height are required to confirm our results.

## Data availability statement

The original contributions presented in the study are included in the article/supplementary material. Further inquiries can be directed to the corresponding author.

## Ethics statement

The studies involving humans were approved by Institutional Review Board of the Faculty of Medicine, Chulalongkorn University (IRB No.0726/66). The studies were conducted in accordance with the local legislation and institutional requirements. Written informed consent for participation was not required from the participants or the participants’ legal guardians/next of kin because data collection from medical record: Retrospective study.

## Author contributions

KS: Writing – review & editing, Conceptualization, Formal analysis, Methodology, Project administration, Supervision, Validation. TT: Conceptualization, Data curation, Formal analysis, Writing – original draft. SA: Investigation, Writing – original draft. NN: Methodology, Project administration, Writing – original draft. VS: Supervision, Writing – review & editing. SW: Supervision, Writing – review & editing.
